# A Genome-Wide Association Study for Hypertensive Kidney Disease in Korean Men

**DOI:** 10.3390/genes12050751

**Published:** 2021-05-17

**Authors:** Hye-Rim Kim, Hyun-Seok Jin, Yong-Bin Eom

**Affiliations:** 1 Department of Medical Sciences, Graduate School, Soonchunhyang University, Asan 31538, Chungnam, Korea; goa6471@naver.com; 2 Department of Biomedical Laboratory Science, College of Life and Health Sciences, Hoseo University, Asan 31499, Chungnam, Korea; jinhs@hoseo.edu; 3 Department of Biomedical Laboratory Science, College of Medical Sciences, Soonchunhyang University, Asan 31538, Chungnam, Korea

**Keywords:** hypertensive kidney disease (HKD), estimated glomerular filtration rate (eGFR), *FANCM* gene, single nucleotide polymorphism (SNP)

## Abstract

Hypertension is one of the major risk factors for chronic kidney disease (CKD), and the coexistence of hypertension and CKD increases morbidity and mortality. Although many genetic factors have been identified separately for hypertension and kidney disease, studies specifically focused on hypertensive kidney disease (HKD) have been rare. Therefore, this study aimed to identify loci or genes associated with HKD. A genome-wide association study (GWAS) was conducted using two Korean cohorts, the Health Examinee (HEXA) and Korean Association REsource (KARE). Consequently, 19 single nucleotide polymorphisms (SNPs) were found to be significantly associated with HKD in the discovery and replication phases (*p* < 5 × 10^−8^, *p* < 0.05, respectively). We further analyzed HKD-related traits such as the estimated glomerular filtration rate (eGFR), creatinine, blood urea nitrogen (BUN), systolic blood pressure (SBP) and diastolic blood pressure (DBP) at the 14q21.2 locus, which showed a strong linkage disequilibrium (LD). Expression quantitative trait loci (eQTL) analysis was also performed to determine whether HKD-related SNPs affect gene expression changes in glomerular and arterial tissues. The results suggested that the *FANCM* gene may affect the development of HKD through an integrated analysis of eQTL and GWAS and was the most significantly associated candidate gene. Taken together, this study indicated that the *FANCM* gene is involved in the pathogenesis of HKD. Additionally, our results will be useful in prioritizing other genes for further experiments.

## 1. Introduction

Chronic kidney disease (CKD), which gradually impairs kidney function, is a serious health problem worldwide [1,2]. Hypertension, the persistent state of high blood pressure, in the arteries around the kidneys causes them to narrow and weaken, and then harden [3]. These damaged arteries fail to deliver enough blood to the kidneys, which are highly dependent on an adequate blood supply [4]. Consequently, hypertension is known to be a major risk factor for kidney disease [5]. According to the report from the World Health Organization (WHO), hypertension is a common chronic disease that affects approximately 1.13 billion people (almost one in four men and one in five women) worldwide (https://www.who.int/, accessed on 10 May 2021). Hypertension is a major cause of CKD and increases the prevalence of CKD [6,7,8]. The Korea Disease Control and Prevention Agency (KDCA) published that the prevalence of CKD in 2019 was 9.3% (https://health.kdca.go.kr/, accessed on 10 May 2021). In addition, 99% of CKD patients were reported to have a history of hypertension in the KNOW-CKD cohort. As such, the coexistence of hypertension and CKD is associated with increased morbidity and mortality from cardiovascular disease, the most common cause of death for CKD [8,9].

Increased renin-angiotensin-aldosterone system (RAAS) activity is closely related to the progression of CKD [10]. Angiotensin II (Ang II) overexpression constricts blood vessels and increases blood pressure, and increases tubular sodium reabsorption by stimulating the release of aldosterone [8,11]. In turn, the abnormal activity of RAAS promotes kidney disease and high blood pressure. Interestingly, a previous study demonstrated the association between genetic variants in RAAS-related genes and hypertension in Koreans [12]. Furthermore, several studies have reported that patients with a family history of CKD have an increased risk of developing hypertensive kidney disease (HKD) [13,14,15]. Therefore, since the occurrence of HKD is affected by genetic factors, identifying candidate genes associated with it will provide insights into the etiology.

Genome-wide association studies (GWASs) have identified several candidate loci and genes for hypertension and kidney disease in diverse populations, including Asian, European and African [16,17,18,19,20]. These studies have found numerous single nucleotide polymorphisms (SNPs) associated with CKD or hypertension. Moreover, a recent review highlighted that genes such as *APOL-1*, *DAB2*, *MYH9*, *RAB38*, *SHROOM3* and *UMOD* were involved in hypertension and kidney disease in human and rodent studies [14]. Although many GWAS studies have been performed for specific pathologies related to kidney disease and for hypertension, GWAS data for kidney disease with hypertension are relatively limited.

This study performed a GWAS analysis using a large-scale cohort (HEXA, Health Examinee) to identify loci and genes associated with HKD. The GWAS results were then validated using the Korean Association REsource (KARE) cohort consisting of independent Korean men. As a result, we found a total of 19 SNPs located in chromosomes 2, 4, 13 and 14. Among them, the 14q21.2 position was selected as a candidate locus, and we further analyzed the association between the SNPs in the *PRPF39*, *FKBP3* and *FANCM* genes and the HKD-related traits. From these analyses, we propose that the *FANCM* gene in chromosome 14 is a likely candidate gene for HKD.

## 2. Materials and Methods

### 2.1. Study Participants

This study used two independent cohorts for case-control analysis of HKD. The Health Examinee (HEXA) cohort was used for the discovery phase. Briefly, a total of 173,208 participants aged 40–79 years were recruited from the HEXA cohort as part of the Korean Genome and Epidemiology Study (KoGES). As a result of checking which participants were analyzed with the Korea Biobank array (KoreanChip), genotypes of 58,700 participants were available. Because the prevalence of hypertension and kidney disease differs according to gender, the analysis focused on men (*n* = 20,293) [21,22]. The replication phase consisted of male adults in the Korean Association REsource (KARE) cohort of the KoGES. A total of 4182 male participants aged 40–69 years were selected for this study. A detailed description of the HEXA and KARE cohorts has been reported elsewhere [23]. The present study was approved by the Institutional Review Board (IRB) of the KDCA (KBN-2021-003) and Soonchunhyang University (202012-BR-086-01). Additionally, all participants provided written informed consent.

The basic characteristics of the participants in this study are described in Table 1. Body mass index (BMI) was calculated as weight (kg) per height squared (m^2^). The estimated glomerular filtration rate (eGFR) was calculated based on the Modification of Diet Renal Disease Study (MDRD) formula: eGFR (mL/min/1.72 m^2^) = 186 × serum creatinine^−1.154^ × age^−0.203^. The blood pressure was measured three times in a sitting position by mercury sphygmomanometer, and the average value of measured blood pressures was used. Blood was collected from all participants to measure serum creatinine and blood urea nitrogen (BUN) levels. Serum creatinine was measured using the Jaffe method. For case-control analysis, patients with HKD (cases, *n* = 310 for the HEXA study; *n* = 43 for the KARE study) were defined as follows: systolic blood pressure (SBP) of ≥140 mmHg and/or a diastolic blood pressure (DBP) of ≥90 mmHg, an eGFR of <60 mL/min/1.72 m^2^ and a history of hypertension and kidney disease. Non-HKD (controls, *n* = 2294 for the HEXA study; *n* = 636 for the KARE study) were defined as a SBP of <120 mmHg and a DBP of <80 mmHg, and an eGFR of ≥90 mL/min/1.72 m^2^. Participants diagnosed with hypertension or kidney disease were excluded from the control group. For quantitative analysis, HKD-related phenotypes (eGFR, BUN, serum creatinine, SBP and DBP levels) were further evaluated at the combination phase of the study, which used both HEXA and KARE.

### 2.2. Genotyping

Genomic DNA was extracted from peripheral blood samples of the male participants. The extracted genomic DNA was genotyped using Korea Biobank arrays (KoreanChip). The KoreanChip was developed by the Center for Genome Science at Korea National Institutes of Health (KNIH), using the Affymetrix Axiom^®^ Array (Affymetrix, Santa Clara, CA, USA). A more detailed description of Korean chips has been reported previously in [24]. In each cohort, sample QC was conducted to exclude participants with low genotyping accuracy (<96–99%), high heterozygosity, DNA contamination or gender inconsistency. Additionally, the following markers were removed during the QC process: call rates of <95%, a minor allele frequency of <1% or a *p*-value < 1 × 10^−6^ in the Hardy–Weinberg equilibrium test. IMPUTE v2 software was used for imputation analysis of genotype data with the 1000 Genomes Phase 3 data (reference panel) [24]. The locations of genes were confirmed through the National Center for Biotechnology Information (NCBI) Human Genome Build 37 (hg19).

### 2.3. Statistical Analysis

The GWAS was conducted using PLINK version 1.90 β (https://www.cog-genomics.org/plink2, accessed on 10 May 2021) [25] with the additive genetic model after age adjustment. Statistical significance between cases and controls was determined by Student’s *t*-test. Logistic regression analysis was used to perform the HKD case-control study. Linear regression was used to analyze the association of *FANCM* gene SNPs with HKD quantitative traits. The cutoff *p*-value was *p* < 5 × 10^−5^ in the discovery phase and *p* < 0.05 in the replication phase. Manhattan plots were drawn using the Haploview version 4.1 program (Whitehead Institute for Biomedical Research, Cambridge, MA, USA) to show the GWAS results for male HKD in the discovery and combination phases. LocusZoom website (http://locuszoom.org/, accessed on 10 May 2021) was utilized to draw regional plots. The eQTL analysis was performed using the NephVS eQTL [26] and the Genotype-Tissue Expression (GTEx) [27] databases.

## 3. Results

### 3.1. Participants Characteristics

The clinical characteristics of male participants included in the discovery, replication and combination phases are described in Table 1. Participants were divided into case groups (discovery, *n* = 310; replication, *n* = 43) and control groups (discovery, *n* = 2294; replication, *n* = 636) to perform GWAS between HKD and variants. In the case groups, age, weight and BMI were significantly (*p* < 0.001) higher than the control groups at all phases. The HKD-related traits such as the eGFR, creatinine, BUN and SBP showed statistically significant differences between cases and controls, excluding eGFR in the discovery phase.

### 3.2. Identification of Loci Related to Hypertensive Kidney Disease

**Figure 1:**
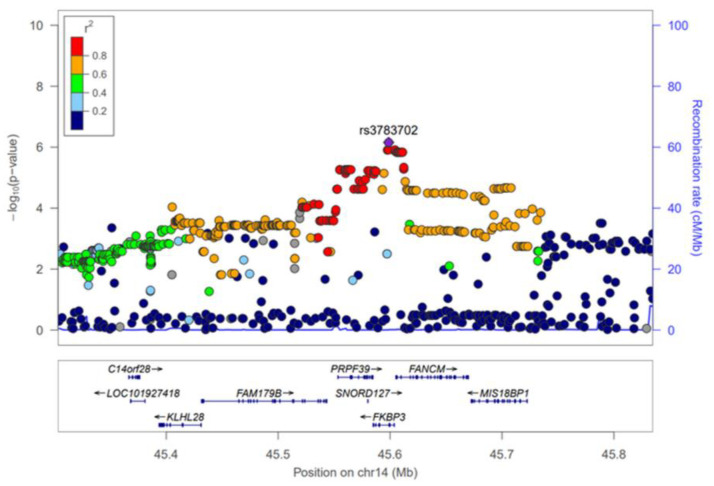
Regional association plot for the 14q21.2. The statistical significance (−log_10_ *p*-value) of the SNPs for the association results with HKD at the combination phase is plotted. The colors show the linkage disequilibrium (*r*^2^) between the SNP with the lowest *p*-value and the remaining SNPs. The genetic recombination rate is shown on the right vertical axis. The plots were generated by the LocusZoom website (http://csg.sph.umich.edu/locuszoom/, accessed on 10 May 2021).

The GWAS results of HKD for the discovery and combination phases were summarized using Manhattan plots (Figure S1) and the SNPs that reached a significance level of *p* < 5 × 10^−5^ in the discovery phase were selected for further analysis. As a result, 352 SNPs were significantly associated with HKD in the discovery phase (Table S1). However, most of these SNPs were not replicated (*p* < 0.05) in the KARE cohort, and of the total, only 19 were replicated (Figure S1, Table 2). The 19 SNPs, which are associated with male HKD, are located on chromosomes 2, 4, 13 and 14. On chromosome 4, rs142696488 in *PABPC4L* was identified as the most significant variant for HKD (OR = 2.69, 95% CI 1.73–4.19, *p* = 1.17 × 10^−5^) in the discovery phase. However, we did not find any evidence for surrounding independent signals at the 4q28.3 locus (Figure S2A). The rs2485016 and rs2485017 near the *LINC00421* gene (13q12.11) yielded statistical significance with HKD (OR = 2.07, 95% CI 1.47–2.90, *p* = 2.96 × 10^−5^, equally) in the discovery phase. The high association of SNPs near the *LINC00421* gene (13q12.11) is represented by a regional plot (Figure S2B). For chromosome 14, 14 SNPs were identified in the *FKBP3* and *FANCM* genes, and of these, nine were in the *FANCM* gene. Therefore, Table 2 shows that SNPs of the *FANCM* gene were most frequently identified as variants associated with HKD. For the most significant SNP, rs3783702, (OR = 1.70, 95% CI 1.33–2.17, *p* = 2.81 × 10^−5^) in the *FKBP3* gene (14q21.2), the regional association plot shows strong linkage disequilibrium (LD) covering the genes *C14orf28, LOC101927418*, *KLHL28*, *FAM179B*, *PRPF39*, *FANCM* and *MIS18BP1* (Figure 1). Although the initial phase analyses had not satisfied the GWAS significance level (*p* < 5 × 10^−8^), replication with the second phase was confirmed, and combined cohort (HEXA + KARE) analyses showed better statistical significance than the initial phase.

### 3.3. Association between FANCM Gene Variants and Hypertensive Kidney Disease-Related Traits

In the combination phase, this study performed an association analysis between 166 SNPs in the candidate genes (*PRPF39*, *FKBP3* and *FANCM*) and HKD and HKD quantitative traits such as eGFR, creatinine, BUN, SBP and DBP (Table S2). Although the range of statistically significant SNPs spanned several genes (Figure 1), the *FANCM* gene was the most likely to be associated with HKD when the functions of each gene and the facts known about each were summarized. Therefore, for SNPs corresponding to the *FANCM* gene region, quantitative analyses were performed on phenotypes related to kidney disease. Of these, 42 SNPs attained statistical significances of *p* < 1 × 10^−5^ and *p* < 0.05 for association with HKD and HKD-related traits, including eGFR and creatinine (Table 3). BUN did not achieve statistical significance (*p* < 0.05) in the 42 SNPs. Participants with minor alleles of these SNPs had an increased risk of HKD, and HKD quantitative traits (eGFR, creatinine) showed tendencies consistent with the risk of HKD. In detail, rs3783702 in the *FKBP3* gene not only shows the highest association with HKD (OR = 1.77, 95% CI 1.41–2.21, *p* = 7.03 × 10^−7^) but also shows a significant association with eGFR (*β* = −0.815, *p* = 9.76 × 10^−5^) and creatinine (*β* = 0.0084, *p* = 1.88 × 10^−5^). The SNPs (rs79911256 and rs78481117) in the *PRPF39* gene had an equally high association with HKD (OR = 1.69, 95% CI 1.35–2.11, *p* = 5.50 × 10^−6^), eGFR (*β* = −0.809, *p* = 1.07 × 10^−4^) and creatinine (*β* = 0.0083, *p* = 2.42 × 10^−5^). Additionally, rs10138997 in the *FANCM* gene, a non-synonymous variant, was significantly associated with HKD (OR = 1.73, 95% CI 1.38–2.16, *p* = 1.48 × 10^−6^), eGFR (*β* = −0.770, *p* = 1.81 × 10^−4^) and creatinine (*β* = 0.0079, *p* = 4.26 × 10^−5^). 

### 3.4. Functional Analysis of FANCM Variants with eQTL

**Figure 2:**
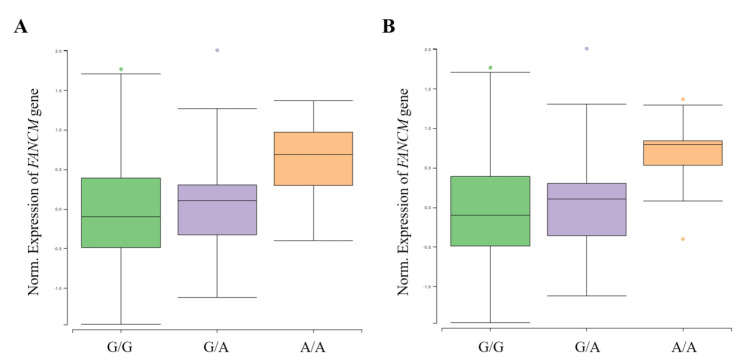
*FANCM* expression for genotypes rs3783702 (**A**) and rs10138997 (**B**) in glomeruli. The data are from the NephQTL database (http://nephqtl.org/, accessed on 10 May 2021). Gene expression for the rs3783702 (*β* = 0.376, *p* = 0.040) and rs10138997 (*β* = 0.389, *p* = 0.027) genotypes in glomeruli were confirmed and were statistically significant. *p*-values were calculated by linear regression.

**Figure 3:**
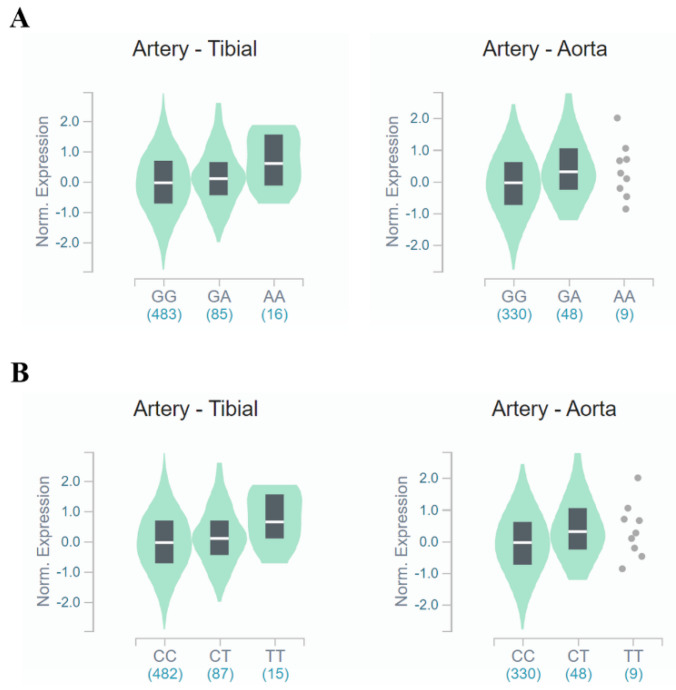
*FANCM* expression for genotypes rs3783702 (**A**) and rs10138997 (**B**) in the arteries. The data are from the GTExPortal. Gene expression for rs3783702 (tibial artery: *β* = 0.204, *p* = 1.2 × 10^−5^, aortic artery: *β* = 0.252, *p* = 6.6 × 10^−5^) and rs10138997 (tibial artery: *β* = 0.211, *p* = 7.1 × 10^−6^, aortic artery: *β* = 0.252, *p* = 6.6 × 10^−5^) genotypes in the arteries was confirmed and were significantly different. *p*-values were calculated by linear regression.

A functional analysis was performed using GTEx (https://www.gtexportal.org/, accessed on 10 May 2021) and NephVS eQTL (http://nephqtl.org/, accessed on 10 May 2021) databases to determine whether HKD-related SNPs affect gene expression changes (Figure 2 and Figure 3). We selected rs3783702, which showed the highest significance at the 14q.21.2 locus, and rs10138997 as the non-synonymous variant, and eQTL analysis was performed using glomerular and arterial tissues associated with HKD. In the glomerulus (Figure 2), the expression levels of the *FANCM* gene were significantly increased in HKD patients possessing the minor alleles of rs3783702 (*β* = 0.376, *p* = 0.040) and rs10138997 (*β* = 0.389, *p* = 0.027).

In tibial and aortic arteries (Figure 3), *FANCM* gene expression was increased (tibial artery: *β* = 0.204, *p* = 1.2 × 10^−5^, aortic artery: *β* = 0.252, *p* = 6.6 × 10^−5^, respectively) for people carrying the minor allele of rs3783702. Likewise, minor allele carriers of rs10138997 increased (tibial artery: *β* = 0.211, *p* = 7.1 × 10^−6^, aortic artery: *β* = 0.252, *p* = 6.6 × 10^−5^, respectively) *FANCM* gene expression in the tibial and aortic arteries. These results demonstrated that expression of the *FANCM* gene in renal glomeruli and arteries was different depending on the genotypes of HKD-related variants.

## 4. Discussion

Impaired kidney function activates the renin-angiotensin-aldosterone system (RAAS), causing the kidneys to produce vasoactive hormones which raise blood pressure [11]. Jin et al. assessed the association between RAAS-related genes with blood pressure and hypertension in a Korean population [12]. As a result, three variants (rs11737660, rs6810951 and rs10519963) in *NR3C2* were associated with both blood pressure and hypertension. Therefore, it has been demonstrated that hypertension is affected by genetic factors of RAAS, which is closely related to CKD. The mechanisms involved in the pathogenesis of HKD are still unclear; however, it is known that the prevalence of HKD is affected by a family history of CKD [28]. Although many GWAS studies have provided strong evidence for genetic variants associated with hypertension and with kidney disease, research on HKD is still limited [16,20,29,30,31]. The prevalence of hypertension and kidney disease differs according to gender [21,22]. A previous study showed that women with hypertension had a relatively 23% lower risk for CKD or end stage renal disease (ESRD) compared to men with hypertension in the integrated analysis of six cohorts [32]. In other words, hypertension in men had a greater risk factor for CKD and ESRD than women. Therefore, this study focused on identifying loci and genes associated with HKD in men using the GWAS approach with two Korean cohorts (HEXA and KARE).

Several loci that reached statistical significance (*p* < 5 × 10^−8^) at the discovery phase were identified, but most of them were not replicated in an independent cohort (Figure S1). Despite this, we were able to identify three novel genetic loci associated with HKD and these were *PABPC4L*, *LINC00421* and *FKBP3*/*FANCM*. The rs142696488 variant of the *PABPC4L* gene indicated the highest significance (discovery, *p* = 1.17 × 10^−5^; combination, *p* = 5.54 × 10^−7^) with HKD, but it did not show a strong LD with surrounding SNPs at the 4q28.3 position (Table 2, Figure S2). The *ANKRD26P3* gene (near *LINC00421* gene) at the 13q12.11 position was associated with post-menopausal osteoporosis [33] and large artery atherosclerosis (LAA) [34]. LAA is a systemic disease that narrows arteries due to risk factors such as hypertension, diabetes and hyperlipidemia [35]. Previous studies have reported that LAA can cause kidney damage [36]. However, Lee et al., who conducted the GWAS on LAA, did not focus on *ANKRD26P3*, which is known as a pseudogene, and only rs2812748 was mentioned [34]; however, this SNP was not observed in Korean genotype data. Moreover, we did not find any other evidence that SNPs identified through GWA analysis (rs2485016 and rs2485017, located downstream of the *LINC00421* gene), or surrounding SNPs (mainly located in the intron of the *ANKRD26P3* gene) with high *r*^2^ values, could function in HKD.

The regional plot of 14q21.2 (chromosome 14 from 45,303,301 to 45,834,804 bp) shows that there are several SNPs in the LD with the top SNP (rs3783702) associated with HKD (Figure 1). This extensive region contains nine genes (Figure 1). Of these, the most promising candidate was the *FANCM* gene. *FANCM* [Fanconi Anemia (FA) complementation group M, OMIM: 609644], which is located in chromosome 14q21.2, is associated with Fanconi Anemia (FA) and Bloom Syndrome (BSyn) diseases [37]. FA and BSyn are rare genetic disorders caused by chromosomal mutations involving abnormal DNA repair and a predisposition towards cancer [38,39]. Previous studies have revealed that FA and BSyn complications are due to abnormal renal structures and type 2 diabetes caused by insulin resistance, respectively [40,41,42,43]. Moreover, Li et al. recently demonstrated that the *FANCI* gene included in the FA complementation group upregulates pulmonary arterial hypertension [44]. In sum, these circumstantially supportive clinical phenotypes strongly support the possibility that the *FANCM* gene is associated with the development of HKD. 

On the one hand, this study has the limitation of a cross-sectional study, not a longitudinal study. Moreover, CKD and hypertension are affected by anti-hypertensive drugs, acid-base status, volume status and several physiological factors, including age, sex, nutrition, exercise, the timing of check-ups, diet and various diseases. Therefore, additional prospective cohort studies are needed to clarify the association between CKD and hypertension. Additionally, further analyses should consider the inclusion of various factors affecting the diseases.

## 5. Conclusions

This study identified several novel loci and genes related to HKD through GWA analysis. Of the four loci, we focused on the 14q.21.1 position, the most promising candidate for HKD risk, and analyzed the association with HKD-related traits such as eGFR, creatinine, BUN, SBP and DBP. Finally, we performed an integrative analysis of eQTL and GWAS results, and collectively, these suggest that the most potent candidate gene associated with HKD is the *FANCM* gene. However, further large-scale studies of other ethnicities are needed to investigate the precise mechanism by which *FANCM* and its gene product are involved in HKD.

## Figures and Tables

**Table 1:** Characteristics of male participants in this study.

Characteristics	Quantitative Trait Analysis (No. of Participants = 22,910)	Discovery (No. of Control = 2294, No. of Case = 310)			Replication (No. of Control = 636, No. of Case = 43)			Combination (No. of Control = 2930, No. of Case = 353)		
		Controls	Cases	*p*-Value *	Controls	Cases	*p*-Value *	Controls	Cases	*p*-Value *
Age (M years ± SD)	54.71 ± 8.51	52.66 ± 7.80	62.06 ± 6.67	**<0.001**	50.52 ± 7.81	56.77 ± 7.92	**<0.001**	52.20 ± 7.85	61.42 ± 7.04	**<0.001**
Height (M cm ± SD)	168.49 ± 5.76	168.88 ± 5.78	167.81 ± 5.75	0.002	166.38 ± 5.88	165.85 ± 5.90	0.564	168.34 ± 5.90	167.57 ± 5.79	0.021
Weight (M kg ± SD)	69.38 ± 9.15	66.30 ± 8.59	71.73 ± 8.90	**<0.001**	65.25 ± 9.25	70.92 ± 9.98	**<0.001**	66.08 ± 8.75	71.63 ± 9.03	**<0.001**
BMI (M kg/m^2^ ± SD)	24.39 ± 2.59	23.22 ± 2.57	25.44 ± 2.67	**<0.001**	23.52 ± 2.76	25.77 ± 3.16	**<0.001**	23.29 ± 2.62	25.48 ± 2.73	**<0.001**
eGFR (ml/min/1.73 m^2^)	88.94 ± 16.06	102.03 ± 10.45	51.34 ± 8.57	0.304	107.81 ± 12.82	73.32 ± 24.49	**<0.001**	103.28 ± 11.26	54.01 ± 13.71	**<0.001**
Creatinine (mg/dl)	0.96 ± 0.15	0.85 ± 0.07	1.40 ± 0.10	0.002	0.82 ± 0.08	1.21 ± 0.39	**<0.001**	0.84 ± 0.07	1.37 ± 0.19	**<0.001**
BUN (mg/dl)	15.26 ± 3.77	14.49 ± 3.57	20.65 ± 4.34	**<0.001**	14.64 ± 3.41	16.99 ± 4.15	**<0.001**	14.52 ± 3.53	20.17 ± 4.48	**<0.001**
SBP (mmHg)	125.06 ± 14.42	109.42 ± 7.01	129.14 ± 14.36	**<0.001**	110.83 ± 9.09	136.47 ± 17.86	**<0.001**	109.73 ± 7.53	130.04 ± 15.00	**<0.001**
DBP (mmHg)	78.66 ± 9.74	68.44 ± 5.89	78.97 ± 9.45	**<0.001**	73.92 ± 6.41	89.49 ± 10.12	**<0.001**	69.63 ± 6.42	80.26 ± 10.12	**<0.001**
Proteinuria (%)				**<0.001**			0.003			**<0.001**
−/+	3115 (96.9)	2,206 (98.7)	248 (82.7)		623 (99.5)	38 (90.5)		2829 (98.5)	286 (83.6)	
1~2+	88 (2.7)	28 (12.5)	43 (14.3)		13 (2.1)	4 (9.5)		41 (1.4)	47 (13.7)	
3~4+	10 (0.3)	1 (0.04)	9 (3.0)		-	-		1 (0.03)	9 (2.6)	
Hematuria (%)				**<0.001**			0.032			**<0.001**
−/+	3098 (95.0)	2184 (95.6)	264 (88.0)		612 (96.2)	38 (90.5)		2796 (95.8)	302 (88.3)	
1~2+	142 (4.4)	89 (3.9)	28 (9.3)		22 (3.5)	3 (7.1)		111 (3.8)	31 (9.1)	
3~4+	22 (0.7)	11 (0.5)	8 (2.7)		2 (0.3)	1 (2.4)		13 (0.4)	9 (2.6)	

BMI, body mass index; eGFR, estimated glomerular filtration rate; BUN, blood urea nitrogen; SBP, systolic blood pressure; DBP, diastolic blood pressure; M, mean value; SD, standard deviation. * Significant differences between the cases and controls were determined by Student’s *t*-test.

**Table 2:** Loci associated with hypertensive kidney disease in Korean men.

No.	SNP	Nearest Gene	Chr:Position	Minor Allele	Function	Discovery MAF		Discovery (No. of Controls = 2294, No. of Cases = 310)		Replication MAF		Replication (No. of Controls = 636, No. of Cases = 43)		Combination MAF		Combination (No. of Controls = 2930, No. of Cases = 353)	
						Controls	Cases	OR (95%CI)	*p*-Value	Controls	Cases	OR (95%CI)	*p*-Value	Controls	Cases	OR (95%CI)	*p*-Value
1	rs146679300	-	2:46888508	A	-	0.031	0.058	2.48 (1.62–3.78)	2.60 × 10^−5^	0.038	0.081	2.94 (1.20–7.22)	0.019	0.032	0.061	2.54 (1.74–3.72)	1.64 × 10^−6^
2	rs146197671	-	4:134816935	T	-	0.026	0.053	2.62 (1.68–4.09)	2.34 × 10^−5^	0.030	0.070	3.16 (1.24–8.05)	0.016	0.027	0.055	2.73 (1.82–4.08)	1.04 × 10^−6^
3	rs142696488	*PABPC4L*	4:134958651	C	Intron	0.029	0.055	2.69 (1.73–4.19)	1.17 × 10^−5^	0.030	0.070	3.11 (1.22–7.93)	0.017	0.029	0.057	2.78 (1.86–4.14)	**5.54 × 10^−7^**
4	rs2485016	*LINC00421*	13:19921380	C	Downstream	0.055	0.097	2.07 (1.47–2.90)	2.96 × 10^−5^	0.059	0.116	2.31 (1.12–4.75)	0.023	0.056	0.099	2.11 (1.55–2.86)	1.95 × 10^−6^
5	rs2485017	*LINC00421*	13:19921382	G	Downstream	0.055	0.097	2.07 (1.47–2.90)	2.96 × 10^−5^	0.059	0.116	2.31 (1.12–4.75)	0.023	0.056	0.099	2.11 (1.55–2.86)	1.95 × 10^−6^
6	rs28637857	*FKBP3*	14:45597638	T	Intron	0.123	0.187	1.67 (1.31–2.14)	4.65 × 10^−5^	0.141	0.221	2.03 (1.16–3.56)	0.013	0.127	0.191	1.74 (1.39–2.18)	1.25 × 10^−6^
7	rs3783702	*FKBP3*	14:45598754	A	Intron	0.121	0.187	1.70 (1.33–2.17)	2.81 × 10^−5^	0.140	0.221	2.04 (1.17–3.58)	0.013	0.125	0.191	1.77 (1.41–2.21)	**7.03 × 10^−7^**
8	rs3783700	*FKBP3*	14:45598870	C	Intron	0.123	0.187	1.67 (1.31–2.14)	4.65 × 10^−5^	0.140	0.221	2.04 (1.16–3.57)	0.013	0.127	0.191	1.74 (1.39–2.18)	1.23 × 10^−6^
9	rs3825625	*FANCM, FKBP3*	14:45604404	T	Upstream	0.130	0.192	1.68 (1.32–2.15)	3.21 × 10^−5^	0.146	0.221	1.94 (1.10–3.41)	0.021	0.133	0.196	1.73 (1.39–2.17)	1.35 × 10^−6^
10	rs8009193	*FANCM, FKBP3*	14:45604461	T	Upstream	0.130	0.192	1.69 (1.32–2.16)	2.95 × 10^−5^	0.146	0.221	1.94 (1.10–3.41)	0.021	0.133	0.196	1.74 (1.39–2.18)	1.23 × 10^−6^
11	rs2033385	*FANCM*	14:45605818	T	Intron	0.130	0.192	1.68 (1.31–2.14)	3.52 × 10^−5^	0.146	0.221	1.94 (1.10–3.41)	0.021	0.133	0.196	1.73 (1.38–2.16)	1.48 × 10^−6^
12	rs10138997	*FANCM*	14:45606287	T	Non-synonymous(S175F)	0.130	0.192	1.68 (1.31–2.14)	3.52 × 10^−5^	0.146	0.221	1.94 (1.10–3.41)	0.021	0.133	0.196	1.73 (1.38–2.16)	1.48 × 10^−6^
13	rs73340655	*FANCM*	14:45607386	T	Intron	0.130	0.192	1.68 (1.31–2.14)	3.52 × 10^−5^	0.145	0.221	1.94 (1.11–3.41)	0.021	0.133	0.196	1.73 (1.39–2.16)	1.46 × 10^−6^
14	rs73340659	*FANCM*	14:45607524	A	Intron	0.130	0.192	1.68 (1.31–2.14)	3.52 × 10^−5^	0.146	0.221	1.94 (1.10–3.41)	0.021	0.133	0.196	1.73 (1.38–2.16)	1.48 × 10^−6^
15	rs28370281	*FANCM*	14:45608107	C	Intron	0.130	0.192	1.68 (1.31–2.14)	3.52 × 10^−5^	0.146	0.221	1.94 (1.10–3.41)	0.021	0.133	0.196	1.73 (1.38–2.16)	1.48 × 10^−6^
16	rs78907363	*FANCM*	14:45609190	C	Intron	0.130	0.192	1.68 (1.31–2.14)	3.52 × 10^−5^	0.146	0.221	1.94 (1.10–3.41)	0.021	0.133	0.196	1.73 (1.38–2.16)	1.48 × 10^−6^
17	rs8017844	*FANCM*	14:45609669	G	Intron	0.130	0.192	1.68 (1.31–2.14)	3.52 × 10^−5^	0.146	0.221	1.94 (1.10–3.41)	0.021	0.133	0.196	1.73 (1.38–2.16)	1.48 × 10^−6^
18	rs112588324	*FANCM*	14:45611083	C	Intron	0.130	0.192	1.68 (1.31–2.14)	3.52 × 10^−5^	0.146	0.221	1.94 (1.10–3.41)	0.021	0.133	0.196	1.73 (1.38–2.16)	1.48 × 10^−6^
19	rs117640296	*FANCM*	14:45611086	A	Intron	0.130	0.192	1.68 (1.31–2.14)	3.52 × 10^−5^	0.145	0.221	1.94 (1.11–3.41)	0.021	0.133	0.196	1.73 (1.39–2.16)	1.46 × 10^−6^

SNP, single nucleotide polymorphism; Chr, chromosome; MAF, minor allele frequency; OR, odds ratio; CI, confidence interval. Odds ratios were calculated after adjusting for age.

**Table 3:** Results of an association analysis between SNPs in the *PRPF39*, *FKBP3* and *FANCM* genes and hypertensive kidney disease and kidney function-related traits.

Nearest Gene	SNP	Minor Allele	MAF	Function	HKD		eGFR		Creatinine		BUN	
					OR (95%CI)	*p*-Value	*β* ± S.E.	*p*-Value	*β* ± S.E.	*p*-Value	*β* ± S.E.	*p*-Value
*PRPF39*	**rs79911256**	T	0.184	Intron	1.69 (1.35–2.11)	5.50 × 10^−6^	−0.809 ± 0.209	1.07 × 10^−4^	0.0083 ± 0.0020	2.42 × 10^−5^	0.063 ± 0.049	0.203
*PRPF39*	rs17115809	G	0.184	Intron	1.69 (1.35–2.11)	5.57 × 10^−6^	−0.802 ± 0.209	1.22 × 10^−4^	0.0082 ± 0.0020	2.75 × 10^−5^	0.063 ± 0.049	0.203
*PRPF39*	**rs78481117**	G	0.184	Intron	1.69 (1.35–2.11)	5.50 × 10^−6^	−0.809 ± 0.209	1.07 × 10^−4^	0.0083 ± 0.0020	2.42 × 10^−5^	0.063 ± 0.049	0.203
*PRPF39*	rs58826926	G	0.184	Intron	1.69 (1.35–2.11)	5.57 × 10^−6^	−0.802 ± 0.209	1.22 × 10^−4^	0.0082 ± 0.0020	2.75 × 10^−5^	0.063 ± 0.049	0.203
*PRPF39*	rs73349842	G	0.184	Intron	1.69 (1.35–2.11)	5.50 × 10^−6^	−0.802 ± 0.209	1.24 × 10^−4^	0.0082 ± 0.0020	2.78 × 10^−5^	0.062 ± 0.049	0.210
*PRPF39*	rs79059197	T	0.184	Intron	1.67 (1.34–2.09)	7.32 × 10^−6^	−0.797 ± 0.209	1.32 × 10^−4^	0.0082 ± 0.0020	2.93 × 10^−5^	0.060 ± 0.049	0.224
*PRPF39*	rs10143806	A	0.184	Intron	1.68 (1.34–2.11)	6.20 × 10^−6^	−0.809 ± 0.209	1.08 × 10^−4^	0.0082 ± 0.0020	2.47 × 10^−5^	0.067 ± 0.049	0.171
*PRPF39*	rs57003561	C	0.184	Intron	1.69 (1.35–2.11)	5.57 × 10^−6^	−0.802 ± 0.209	1.22 × 10^−4^	0.0082 ± 0.0020	2.75 × 10^−5^	0.063 ± 0.049	0.203
*PRPF39*	rs73349853	T	0.184	Intron	1.69 (1.35–2.11)	5.50 × 10^−6^	−0.802 ± 0.209	1.24 × 10^−4^	0.0082 ± 0.0020	2.78 × 10^−5^	0.062 ± 0.049	0.210
*PRPF39*	rs17115811	A	0.184	Intron	1.69 (1.35–2.11)	5.57 × 10^−6^	−0.802 ± 0.209	1.22 × 10^−4^	0.0082 ± 0.0020	2.75 × 10^−5^	0.063 ± 0.049	0.203
*PRPF39*	rs73349857	T	0.184	Intron	1.69 (1.35–2.11)	5.57 × 10^−6^	−0.802 ± 0.209	1.22 × 10^−4^	0.0082 ± 0.0020	2.75 × 10^−5^	0.063 ± 0.049	0.203
*PRPF39*	rs1311177170	CT	0.205	Intron	1.65 (1.33–2.05)	5.36 × 10^−6^	−0.700 ± 0.198	4.15 × 10^−4^	0.0070 ± 0.0019	1.80 × 10^−4^	0.055 ± 0.047	0.237
*PRPF39*	rs117107185	A	0.184	Intron	1.69 (1.35–2.11)	5.50 × 10^−6^	−0.804 ± 0.209	1.20 × 10^−4^	0.0082 ± 0.0020	2.63 × 10^−5^	0.061 ± 0.049	0.215
*PRPF39*	rs73349858	C	0.184	Intron	1.69 (1.35–2.11)	5.50 × 10^−6^	−0.796 ± 0.209	1.38 × 10^−4^	0.0082 ± 0.0020	3.01 × 10^−5^	0.060 ± 0.049	0.221
*PRPF39*	rs74244132	G	0.184	Intron	1.69 (1.35–2.11)	5.50 × 10^−6^	−0.796 ± 0.209	1.38 × 10^−4^	0.0082 ± 0.0020	3.01 × 10^−5^	0.060 ± 0.049	0.221
*PRPF39*	rs59353994	G	0.187	Intron	1.68 (1.34–2.10)	5.89 × 10^−6^	−0.742 ± 0.206	3.16 × 10^−4^	0.0077 ± 0.0019	6.59 × 10^−5^	0.066 ± 0.049	0.172
*PRPF39*	rs73340622	G	0.187	Intron	1.67 (1.33–2.09)	7.29 × 10^−6^	−0.731 ± 0.205	3.77 × 10^−4^	0.0076 ± 0.0019	8.05 × 10^−5^	0.067 ± 0.048	0.168
*PRPF39*	rs73340626	C	0.187	Intron	1.68 (1.34–2.10)	5.89 × 10^−6^	−0.749 ± 0.206	2.78 × 10^−4^	0.0078 ± 0.0019	5.68 × 10^−5^	0.067 ± 0.049	0.165
*FKBP3*	rs2016737	T	0.187	Intron	1.68 (1.34–2.10)	5.97 × 10^−6^	−0.749 ± 0.206	2.74 × 10^−4^	0.0078 ± 0.0019	5.63 × 10^−5^	0.068 ± 0.049	0.160
*FKBP3*	rs2016738	G	0.187	Intron	1.67 (1.33–2.09)	7.78 × 10^−6^	−0.765 ± 0.206	2.06 × 10^−4^	0.0079 ± 0.0019	4.30 × 10^−5^	0.066 ± 0.049	0.175
*FKBP3*	rs8022499	T	0.189	Intron	1.67 (1.34–2.09)	6.52 × 10^−6^	−0.623 ± 0.203	2.16 × 10^−3^	0.0067 ± 0.0019	3.94 × 10^−4^	0.063 ± 0.048	0.185
*FKBP3*	rs111329832	C	0.187	Intron	1.68 (1.34–2.10)	5.89 × 10^−6^	−0.749 ± 0.206	2.78 × 10^−4^	0.0078 ± 0.0019	5.68 × 10^−5^	0.067 ± 0.049	0.165
*FKBP3*	rs76410513	C	0.187	Intron	1.68 (1.34–2.10)	6.25 × 10^−6^	−0.758 ± 0.206	2.30 × 10^−4^	0.0078 ± 0.0019	4.87 × 10^−5^	0.068 ± 0.049	0.164
*FKBP3*	rs75451707	A	0.192	Intron	1.66 (1.33–2.07)	7.22 × 10^−6^	−0.741 ± 0.204	2.77 × 10^−4^	0.0073 ± 0.0019	1.25 × 10^−4^	0.053 ± 0.048	0.268
*FKBP3*	rs28637857	T	0.187	Intron	1.74 (1.39–2.18)	1.25 × 10^−6^	−0.768 ± 0.209	2.32 × 10^−4^	0.0079 ± 0.0020	4.92 × 10^−5^	0.062 ± 0.049	0.208
*FKBP3*	rs3783702	A	0.187	Intron	1.77 (1.41–2.21)	7.03 × 10^−7^	−0.815 ± 0.209	9.76 × 10^−5^	0.0084 ± 0.0020	1.88 × 10^−5^	0.058 ± 0.049	0.238
*FKBP3*	rs3783700	C	0.187	Intron	1.74 (1.39–2.18)	1.23 × 10^−6^	−0.775 ± 0.209	2.05 × 10^−4^	0.0080 ± 0.0020	4.34 × 10^−5^	0.062 ± 0.049	0.209
*FANCM, FKBP3*	rs3825625	T	0.192	Upstream	1.73 (1.39–2.17)	1.35 × 10^−6^	−0.771 ± 0.206	1.77 × 10^−4^	0.0079 ± 0.0019	4.26 × 10^−5^	0.049 ± 0.048	0.311
*FANCM, FKBP3*	rs8009193	T	0.192	Upstream	1.74 (1.39–2.18)	1.23 × 10^−6^	−0.775 ± 0.206	1.66 × 10^−4^	0.0079 ± 0.0019	4.20 × 10^−5^	0.049 ± 0.049	0.316
*FANCM*	rs2033385	T	0.192	Intron	1.73 (1.38–2.16)	1.48 × 10^−6^	−0.767 ± 0.206	1.90 × 10^−4^	0.0078 ± 0.0019	4.62 × 10^−5^	0.049 ± 0.048	0.315
*FANCM*	**rs10138997**	T	0.192	Non-synonymous(S175F)	1.73 (1.38–2.16)	1.48 × 10^−6^	−0.770 ± 0.206	1.81 × 10^−4^	0.0079 ± 0.0019	4.26 × 10^−5^	0.050 ± 0.048	0.304
*FANCM*	rs73340655	T	0.192	Intron	1.73 (1.39–2.16)	1.46 × 10^−6^	−0.766 ± 0.206	1.97 × 10^−4^	0.0078 ± 0.0019	4.80 × 10^−5^	0.050 ± 0.048	0.305
*FANCM*	rs73340659	A	0.192	Intron	1.73 (1.38–2.16)	1.48 × 10^−6^	−0.766 ± 0.206	1.94 × 10^−4^	0.0078 ± 0.0019	4.76 × 10^−5^	0.051 ± 0.048	0.296
*FANCM*	rs28370281	C	0.192	Intron	1.73 (1.38–2.16)	1.48 × 10^−6^	−0.766 ± 0.206	1.94 × 10^−4^	0.0078 ± 0.0019	4.76 × 10^−5^	0.051 ± 0.048	0.296
*FANCM*	rs78907363	C	0.192	Intron	1.73 (1.38–2.16)	1.48 × 10^−6^	−0.766 ± 0.206	1.94 × 10^−4^	0.0078 ± 0.0019	4.76 × 10^−5^	0.051 ± 0.048	0.296
*FANCM*	rs8017844	G	0.192	Intron	1.73 (1.38–2.16)	1.48 × 10^−6^	−0.766 ± 0.206	1.94 × 10^−4^	0.0078 ± 0.0019	4.76 × 10^−5^	0.051 ± 0.048	0.296
*FANCM*	rs1254055944	TCCTCCC	0.142	Intron	1.79 (1.40–2.30)	4.51 × 10^−6^	−0.745 ± 0.237	0.002	0.0074 ± 0.0022	8.11 × 10^−4^	0.106 ± 0.056	0.059
*FANCM*	rs112588324	C	0.192	Intron	1.73 (1.38–2.16)	1.48 × 10^−6^	−0.766 ± 0.206	1.94 × 10^−4^	0.0078 ± 0.0019	4.76 × 10^−5^	0.051 ± 0.048	0.296
*FANCM*	rs117640296	A	0.192	Intron	1.73 (1.39–2.16)	1.46 × 10^−6^	−0.773 ± 0.206	1.71 × 10^−4^	0.0079 ± 0.0019	4.21 × 10^−5^	0.051 ± 0.049	0.297
*FANCM*	rs10130368	C	0.192	Intron	1.67 (1.34–2.09)	5.24 × 10^−6^	−0.722 ± 0.206	4.41 × 10^−4^	0.0075 ± 0.0019	1.01 × 10^−4^	0.051 ± 0.048	0.293
*FANCM*	rs10130377	G	0.192	Intron	1.67 (1.34–2.09)	5.24 × 10^−6^	−0.726 ± 0.205	4.14 × 10^−4^	0.0075 ± 0.0019	9.40 × 10^−5^	0.055 ± 0.048	0.253
*FANCM*	rs10141474	A	0.192	Intron	1.68 (1.35–2.09)	4.59 × 10^−6^	−0.727 ± 0.206	4.04 × 10^−4^	0.0075 ± 0.0019	9.22 × 10^−5^	0.055 ± 0.048	0.255

SNP, single nucleotide polymorphism; CHR, chromosome; MAF, minor allele frequency; HKD, hypertensive kidney disease; eGFR, estimated glomerular filtration rate; BUN, blood urea nitrogen; *β*, regression coefficient; S.E., standard error; OR, odds ratio; CI, confidence interval. eGFR, creatinine and BUN, used in the linear regression, were adjusted for age. Odds ratios were calculated after adjusting for age.

## Data Availability

The data presented in this study are available on request from the corresponding author. The data are not publicly available due to ethnical concerns.

## Supplementary Materials

The following are available online at https://www.mdpi.com/article/10.3390/genes12050751/s1, Figure S1: Genome-wide association scan for male hypertensive kidney disease in discovery and combination phases. The vertical axis indicates −log 10 of *p*-value from logistic regression adjusted for age, and the horizontal axis indicates chromosomal positions. The red line shows the threshold (*p* = 5 × 10^−5^). The Manhattan plot was generated by the program Haploview version 4.1. Figure S2: Regional association plots for the 4q28.3 (**A**) and 13q12.11 (**B**) regions. The statistical significance (−log_10_ *p*-value) of the SNPs for the association results with HKD at the combination phase is plotted. The colors show the linkage disequilibrium (*r*^2^) between the top SNP marked with purple diamond and the remaining SNPs. The genetic recombination rates are shown by a blue line. Plots were generated by the LocusZoom website (http://csg.sph.umich.edu/locuszoom/, accessed on 10 May 2021). Table S1: Genetic variants associated with hypertensive kidney disease in discovery phase. Table S2: Results of an association analysis between SNPs in the *PRPF39*, *FKBP3* and *FANCM* genes and hypertensive kidney disease and hypertensive nephropathy-related traits.
