# Supplementary figures and images for: A Genome-Wide Association Study for Hypertensive Kidney Disease in Korean Men
Source: Genes (Basel). 2021 May 17;12(5):751. doi: 10.3390/genes12050751 (PMC8155956; doi:10.3390/genes12050751)

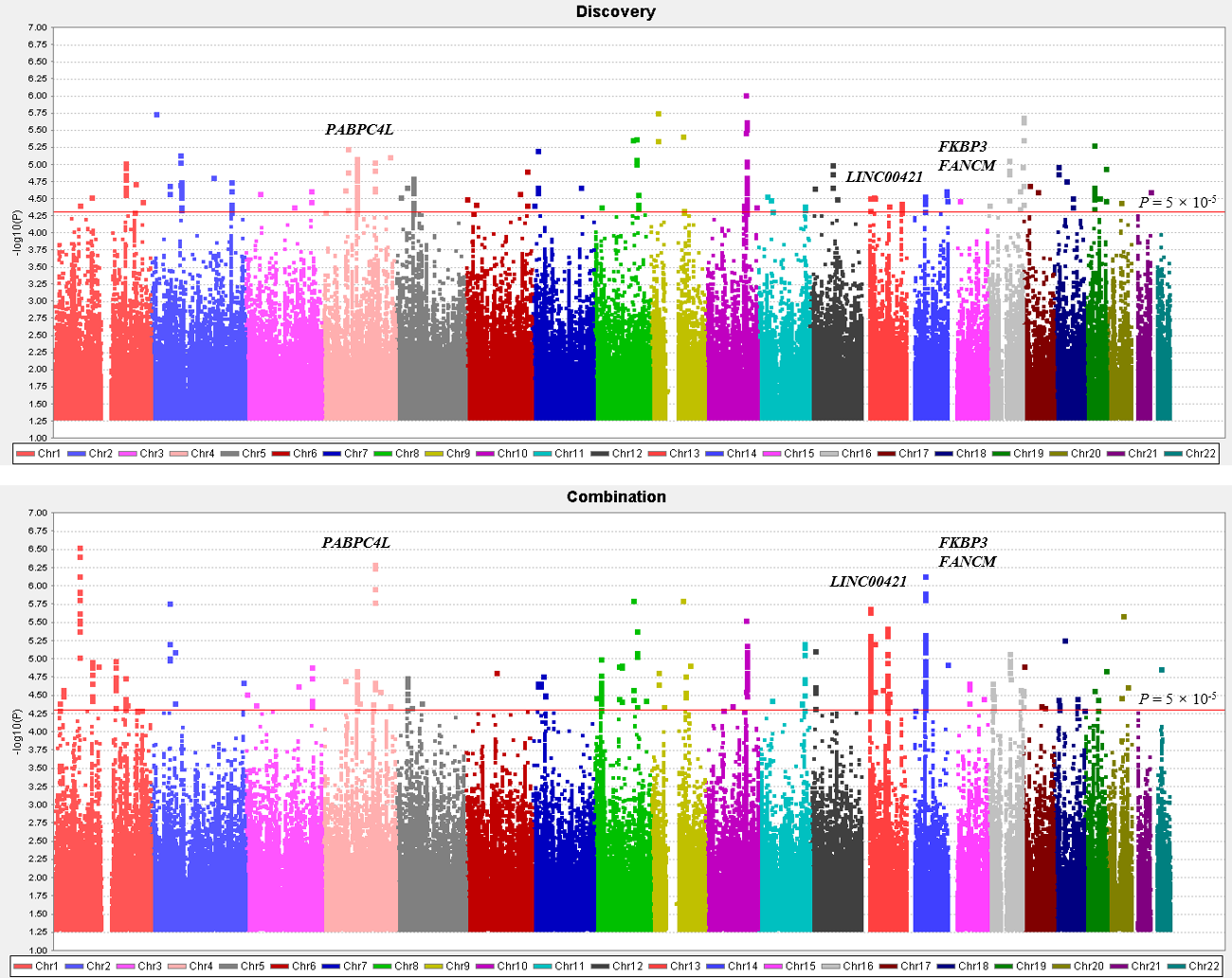

Supplement: Supplementary file 1 [file genes-12-00751-s001.zip › Supplementary Figure 1_600dpi.tif]

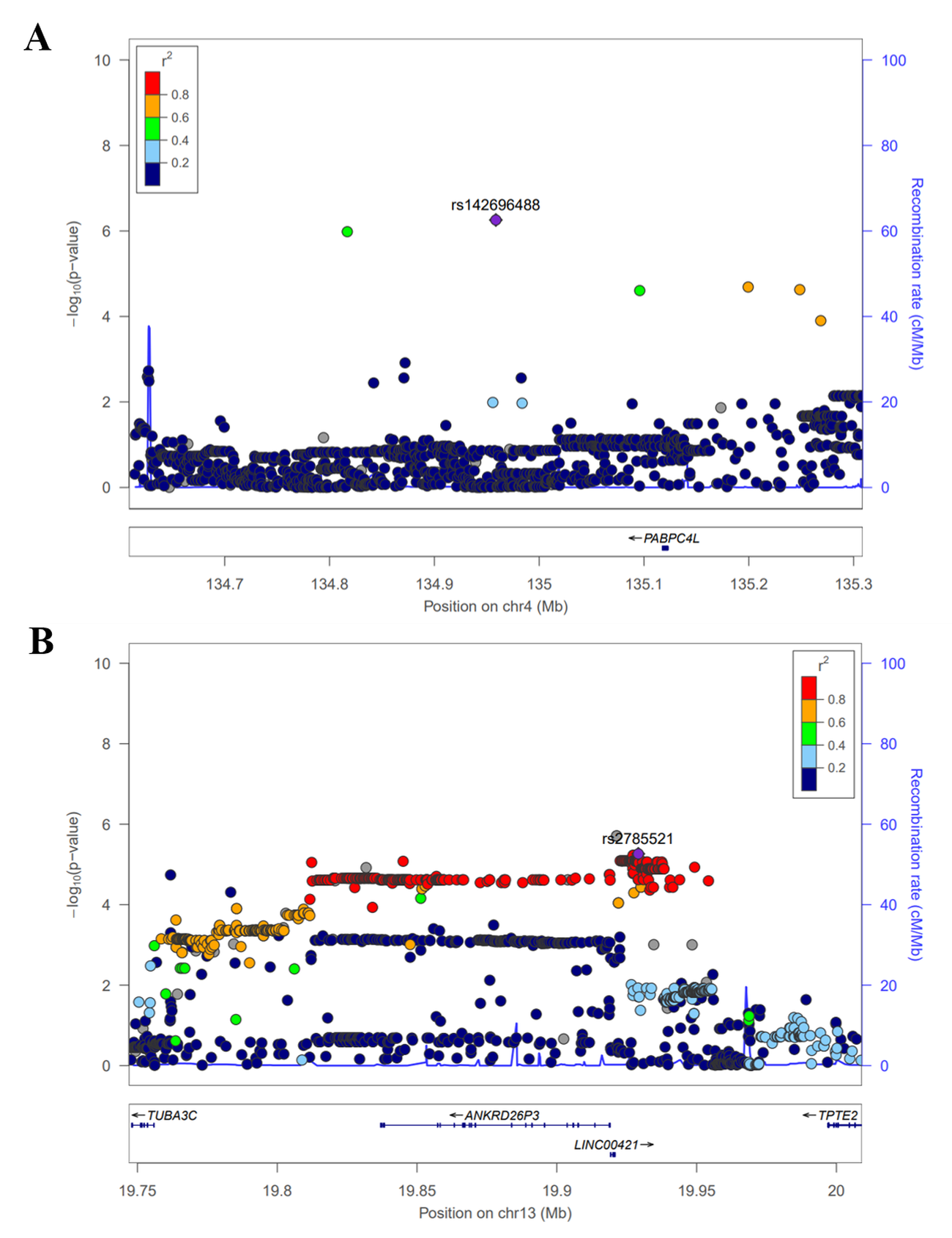

Supplement: Supplementary file 1 [file genes-12-00751-s001.zip › Supplementary Figure 2_600dpi.tif]
